# Complex regional pain syndrome in a competitive athlete and regional osteoporosis assessed by dual-energy X-ray absorptiometry: a case report

**DOI:** 10.1186/1752-1947-8-165

**Published:** 2014-05-27

**Authors:** Karen Hind, Mark I Johnson

**Affiliations:** 1 Carnegie Research Institute, Leeds Metropolitan University, Headingley Campus, Leeds LS6 3QS, UK; 2 Centre for Pain Research, Faculty of Health and Social Sciences, Leeds Metropolitan University, Portland Building, City Campus, Leeds LS1 3HE, UK

**Keywords:** Complex regional pain syndrome, DXA, Exercise, Osteoporosis, Pain

## Abstract

**Introduction:**

Dual-energy X-ray absorptiometry is rarely utilized in the clinical care of patients with complex regional pain syndrome, but may be useful for the non-invasive determination of regional bone fragility and fracture risk, as well as muscular atrophy and regional body composition. This is the first report in the literature of complex regional pain syndrome and musculoskeletal co-morbidities in an athlete, and is the first to focus on dual-energy X-ray absorptiometry for the clinical assessment of complex regional pain syndrome.

**Case presentation:**

In this report, we describe the case of a 29-year-old Caucasian man with type 1 complex regional pain syndrome. His body mass index was 29.4kg/m^2^ at the time of presentation. Despite severe complex regional pain syndrome in the left limb and long term use of a wheelchair, the patient participated in high-performance powerlifting. Dual-energy X-ray absorptiometry revealed marked unilateral differences in bone strength and lean mass between the affected regions and the contralateral regions. Low bone mineral density for age was found in the left hip, with Z-scores ranging from −2.2 to −3.0, and the patient had previously suffered two fractures. Bone density Z-scores in the right hip and legs were normal.

**Conclusions:**

Dual-energy X-ray absorptiometry is a valuable tool for the clinical investigation of musculoskeletal health in patients with complex regional pain syndrome. Regional osteoporosis in complex regional pain syndrome patients is complicated and should be investigated and monitored. Physical activity is possible for some complex regional pain syndrome patients, depending on the type of exercise and the region affected, and it may protect bone density and strength at non affected skeletal sites.

## Introduction

Complex regional pain syndrome (CRPS) can be triggered by peripheral trauma, fracture, surgery, or spontaneously and causes significant functional morbidity. The International Association for the Study of Pain (IASP) [1] defines CRPS type I as “a syndrome that usually develops after an initiating noxious event, is not limited to the distribution of a single peripheral nerve, and is apparently disproportionate to the inciting event. It is associated at some point with evidence of edema, changes in skin blood flow, abnormal sudomotor activity in the region of the pain, or allodynia or hyperalgesia” (p 41).The common symptoms of CRPS include extreme reactions to touch; tremor; pain and temperature; impaired movement; muscle spasms; change in skin color; hair and nail growth; pseudoparalysis, paresis; and autonomic, sensory and vasomotor symptoms [1-4]. The inability to participate in physical activity and a reduced quality of life are consequences of these symptoms. There is no consensus regarding treatment, mainly because of the clinical heterogeneity of CRPS. Common pharmaceutical therapies include pain medication, local anesthetics, intravenous sympathetic blockades, bisphosphonates, calcium channel blockers, spinal cord stimulation and amputation [1,2,4-6].

Of the CRPS comorbidities, region-specific osteoporosis and muscle hypotrophy may also be considered as objective indicators of the disease, although there is a lack of published data on this topic. Osteoporosis is a systemic, skeletal disease characterized by low bone density and microarchitectural deterioration of bone tissue with a consequent increase in bone fragility. Osteoporotic fractures are the clinical endpoints of bone fragility and carry significant mortality and morbidity. Such musculoskeletal entities associated with CRPS [7] are likely to develop in response to disuse due to immobilization and can cause further pain, fracture and disability. It has been reported that CRPS-associated bone loss is characterized by elevated bone turnover and bone resorption [8]. Extensive type 1 and type 2 muscle fiber atrophy, as well as neurogenic myopathy, have also been reported in CRPS patients [9,10]. Dual-energy X-ray absorptiometry (DXA) may be useful for noninvasive, accurate determination of demineralization of bone and fracture risk in CRPS patients. With the recent advances in DXA technology, this tool may also be useful for the determination of CRPS-associated muscular atrophy and unilateral body composition.

## Case presentation

A 29-year-old Caucasian man presented to our institution for DXA investigations. He met the IASP criteria for CRPS and was medically diagnosed with type I CRPS. His symptoms associated with CRPS were first reported at the age of 10 years following orthopedic surgery to the left hip. That surgery was initiated after he had been diagnosed with Calve-Perthes disease at the age of 9 years. Rehabilitation from the surgery was unsuccessful, and he remained wheelchair-bound thereafter because of inability to move his left leg, accompanied by severe, localised pain. There was blue discoloration to the affected limb , and the patient had abnormal hair and toenail growth as well as swelling. His other chronic complaints included severe pain while taking a shower, bruising, and insomnia due to pain. Numerous orthopedic and pediatric physicians confirmed that his Perthes disease was no longer a problem following surgery. The patient had been diagnosed with CRPS at age 13 years. He had fractured his left fifth metatarsal at age 17 years, and he fractured to his left patella at age 24 years. Sensitivity tests conducted at a pain management unit in 2011 revealed marked mechanical allodynia induced by the lightest monofilament (finer than a hair) and hypersensitivity to a pinprick with lasting tingling in the left leg. He received different methods of pain treatment, including guanethidine blocks, which helped only initially for approximately one week. Subsequent treatments included opioids and spinal cord stimulation. Combinations of drugs were prescribed for neuropathic pain, but produced little benefit. The patient had also taken antidepressants (fluoxetine hydrochloride) for several months at age 17 years. At the time of our assessment, the patient was taking amitriptyline, co-codamol and diclofenac.

The long-term symptoms and comorbidities of CRPS can often lead to inability to perform activities of daily living, which usually means that participation in physical activity and high-level sports is not possible. Our patient engaged in regular upper-body resistance exercise training and performed competitively at the international level. He had been participating in the competitive sport of powerlifting for more than three years, despite his severe CRPS symptoms.

The patient was measured while wearing lightweight clothing and no jewelry. His height was measured to the nearest millimeter using a stadiometer (seca United Kingdom, Birmingham, UK), and his body weight was recorded to the nearest 0.1kg with calibrated electronic scales (seca United Kingdom). DXA was performed using a fan-beam Lunar iDXA imager (GE Healthcare, Madison, WI, USA) in standard mode. The machine’s calibration was checked and passed on a daily basis prior to the scanning session using the GE Lunar calibration phantom. There was no significant drift in calibration prior to the scanning session. For the total body scan, the patient was placed in the supine position on the scanning table with his body aligned with the central horizontal axis. His arms were positioned parallel to, but not touching, the body, with his legs fully extended. His feet were not secured with the usual support so as not to cause him any distress. For the total hip scan, the patient was again positioned supine on the scanning table. His arms were placed across his chest, and his feet were placed on either side of the dual femur positioning support. His feet were not strapped, but rather were inwardly rotated to obtain an optimal scan image. The total hip scan also enabled hip structural analysis (HSA) to gain information on the structural geometry and strength of the patient’s left proximal femur. Section modulus, cross-sectional area (CSA (in cm^2^); exclusive of soft-tissue spaces) and cross-sectional moment of inertia results were obtained using HSA. The duration of the total body scan was 7 minutes, and the total hip scan, 2 minutes. The scans were analyzed using the Lunar enCORE software version 13.6 (GE Healthcare).

**Figure 1:**
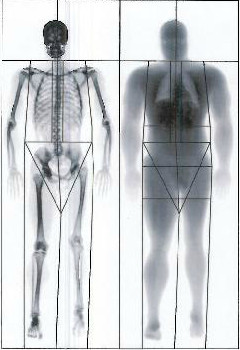
Lunar iDXA image taken from a total body scan of a male athlete with complex regional pain syndrome of the left limb.

The patient’s general results are shown in Table 1. The results for his regional body bone mineral content (BMC) and lean tissue mass (LTM) are shown in Table 2. Figure 1 shows the DXA image of the patient’s total body, with clearly visible unilateral differences in bone and muscle mass. DXA revealed gross muscle wasting of all muscle groups in the left leg. Visual analysis of the scan indicated that his left leg was much shorter than his right leg. Concurrently, the largest unilateral differences in BMC and LTM were found in the legs, with lower mass in the left legs equivalent to 36% and 37% for BMC and LTM, respectively. This did not affect his total body BMD, which was 1.190g/cm^2^, giving an overall normal Z-score of −0.1.

**Table 1:** Anthropometry and bone status

**Characteristics**	**Patient data**
Height	171.4cm
Weight	86.3kg
Body mass index	29.4kg/m^2^
Body fat	26%
Total body bone mineral density Z-score	−0.1
Total hip bone mineral density Z-score	−1.4

**Table 2:** Regional bone mineral content and lean tissue mass derived from total body dual-energy X-ray absorptiometry

	**Bone mineral content (g)**			**Lean tissue mass (kg)**		
**Limb**	**Left**	**Right**	**Difference**	**Left**	**Right**	**Difference**
Arms	264	270	−6	5.3	5.3	0
Trunk	470	559	−89	15.2	14.6	0.5
Legs	331	511	−180	6.7	10.6	−3.9
Total	1,296	1,682	−386	28.8	32.9	−4.1

Data were obtained using a Lunar iDXA (GE Healthcare, Madison, WI, USA).

Table 3 shows the unilateral differences in hip the patient’s BMD and the corresponding Z-scores. The lowest Z-score was −3.0. Differences in BMD between the left and right sides of the hip ranged from 19% to 31% (left < right), and the Z-scores on the left side were −2.2 to −3.0) which indicates low BMD for age according to the International Society for Clinical Densitometry (for men 50 years of age and younger). Table 4 provides the results from the DXA-derived HSA and shows distinct differences in bone geometry between the left and right proximal femurs.

**Table 3:** Unilateral bone mineral density of the total hip and regions of the hip in the patient’s left leg

	**Bone mineral density (g/cm**^**2**^**)**			**Age-matched Z-score**	
**Body region**	**Left**	**Right**	**Difference**	**Left**	**Right**
Femoral neck	0.781	0.965	−0.184	**−2.3**	−0.8
Wards Area	0.634	0.832	−0.199	**−2.6**	−1.0
Trochanter	0.598	0.863	−0.265	**−3.0**	−0.6
Total	0.760	1.055	−0.295	**−2.6**	−0.3

**Table 4:** Unilateral differences in hip structural analysis parameters of the patient’s left proximal femur

**Measurements**	**Left**	**Right**	**Difference**
Section modulus (mm^3^)	802.8	897.7	−94.9
Cross-sectional moment of inertia (mm^4^)	16.8	17.8	−10
Cross-sectional area (mm^2^)	154	179	−25

## Discussion

We recommend the incorporation of DXA measurements in the clinical care of patients with CRPS. Our DXA investigations revealed large bone and LTM differences between the affected and contralateral regions. The bone density in the patient’s left leg and left hip was low, and his bone geometry was compromised, potentially exposing him to greater risk of fragility fracture in the affected regions. Of interest, the patient had also previously had fractures to the left fifth metatarsal and the left patella at ages 17 years and 24 years, respectively. His bone density was normal at non-CRPS sites, which suggests that participation in regular exercise training involving unaffected regions of the body may be of benefit for maintaining bone strength. This case report also demonstrates that participation in high levels of targeted exercise is possible for CRPS patients, depending on the site affected.

DXA is a viable imaging test for CRPS patients both practically and technically, because it is objective and noninvasive, requires minimal patient preparation, can be performed quickly has a high level of accuracy. Minimal patient preparation is particularly important, given the pain often experienced during CRPS clinical investigations. Clinically, DXA can be used to assess for osteoporosis according to the World Health Organization guidelines [11] and provide objective information on asymmetrical regional bone and body composition. Although regional osteoporosis associated with CRPS may be transient, our patient’s low Z-scores and age suggest that bone therapeutic intervention may be of value, especially bearing in mind that he had had CRPS for 19 years with no sign of recovery to date. Our patient was 29 years of age upon presentation to our institution, when he was reaching the end of the peak bone mass accrual period. The failure to attain optimal peak bone mass during childhood and young adulthood increases the risk of osteoporosis and fracture later in life. Patients with osteoporosis may benefit from pharmaceutical interventions to improve bone mass and prevent further bone loss and osteoporotic fracture, which, independently of CRPS, can cause significant pain and disability. Bisphosphonates (risedronate, pamidronate and alendronate) are used widely to treat osteoporosis because of their potent inhibitory effect on bone resorption, and they are now recognized for their analgesic properties in the treatment of CRPS [12,13].

Unfortunately, for our patient, no baseline DXA examination reports of bone density and lean mass prior to or in the early stages of CRPS development were available. This information would have been useful to track how much bone and lean tissue had been lost (or not gained during bone mass accrual with age) through immobilization due to CRPS, the rate of loss, as well as any possible systemic factors. Although it has been suggested that patients with osteoporosis are more susceptible to CRPS [14], the findings in our patient suggest that he acquired regional osteoporosis as a consequence of CRPS rather than vice versa. This was indicated by the marginal unilateral differences in tissue mass between the left and right limbs. Upon referral, longitudinal DXA monitoring of this patient will enable us to gain insights into the progression (or a plateau) of bone loss with the persistence of the disease or bone gain with recovery from CRPS.

## Conclusions

DXA is a valuable tool for the clinical investigation of musculoskeletal health in patients with CRPS. Regional osteoporosis in CRPS patients is complex and should be investigated and monitored. Therapy aimed at improving bone density in affected patients should be considered. Physical activity is not completely out of the question for CRPS patients, depending on the type of exercise and the region affected, and may protect bone density and strength at non-CRPS sites.

## Consent

Written informed consent was obtained from the patient for publication of this case report and any accompanying images. The patient’s sports coach also provided informed consent, and both the patient and his coach were consulted throughout the case report preparation. A copy of the written consent is available for review by the Editor-in-Chief of this journal.

## Abbreviations

BMC: Bone mineral content; BMD: Bone mineral density; BMI: Body mass index; CRPS: Complex regional pain syndrome; CSA: Cross-sectional area; CSMI: Cross-sectional moment of inertia; DXA: Dual-energy X-ray absorptiometry; HSA: Hip structural analysis; IASP: International Association for the Study of Pain; LTM: Lean tissue mass.

## Competing interests

The authors declare they have no competing interests.

## Authors’ contributions

KH collected, analyzed and interpreted the patient DXA data. KH and MJ reviewed the patient’s medical history and prepared the manuscript. Both authors read and approved the final manuscript.

## Authors’ information

KH is a Senior Research Fellow specializing in musculoskeletal physiology and medical imaging. MJ is an expert in the field of pain and analgesia and is a neurophysiologist.
